# Low Protein Diets and Energy Balance: Mechanisms of Action on Energy Intake and Expenditure

**DOI:** 10.3389/fnut.2021.655833

**Published:** 2021-05-13

**Authors:** Adel Pezeshki, Prasanth K. Chelikani

**Affiliations:** ^1^Department of Animal and Food Sciences, Oklahoma State University, Stillwater, OK, United States; ^2^School of Veterinary Medicine, Texas Tech University, Amarillo, TX, United States; ^3^Department of Nutritional Sciences, College of Human Sciences, Texas Tech University, Lubbock, TX, United States

**Keywords:** low protein, food intake, energy expenditure, neuroendocrine, energy balance

## Abstract

Low protein diets are associated with increased lifespan and improved cardiometabolic health primarily in rodents, and likely improve human health. There is strong evidence that moderate to severe reduction in dietary protein content markedly influences caloric intake and energy expenditure, which is often followed by a decrease in body weight and adiposity in animal models. While the neuroendocrine signals that trigger hyperphagic responses to protein restriction are better understood, there is accumulating evidence that increased sympathetic flux to brown adipose tissue, fibroblast growth factor-21 and serotonergic signaling are important for the thermogenic effects of low protein diets. This mini-review specifically focuses on the effect of low protein diets with variable carbohydrate and lipid content on energy intake and expenditure, and the underlying mechanisms of actions by these diets. Understanding the mechanisms by which protein restriction influences energy balance may unveil novel approaches for treating metabolic disorders in humans and improve production efficiency in domestic animals.

## Introduction

Energy balance is a fundamental biological process that is dependent on a complex interplay of calories consumed as macronutrients (carbohydrate, fat, and protein), and energy expended and stored. A dysregulation of the mechanisms that sense and signal dietary nutrients in the gut may predispose to obesity and metabolic complications. Among the macronutrients, the intake of protein is tightly regulated and dietary protein restriction is purported to extend lifespan, improve energy balance and cardiometabolic health (1); however, the underlying mechanisms remain poorly understood. Here we review the potential mechanisms by which dietary protein restriction modulates energy homeostasis to alter energy intake and energy expenditure (EE).

## Regulation Of Food Intake By Low Protein Diets

### Effect of Dietary Protein Restriction on Food Intake

The “protein leverage” hypothesis posits that protein intake is tightly regulated in several species including rodents, small animal pets, birds and humans (2–4). When isocaloric high protein diets are fed, in order to keep the amount of protein consumed constant and avoid protein excess, the total caloric intake and hence the intake of carbohydrates and fats is reduced. As a corollary, when low protein diets are fed, in order to avoid protein deficiency and to meet the protein requirements, the total food consumption is increased and hence the total caloric intake from carbohydrate and fat is also increased as a consequence (2, 3, 5). In humans, diets that are moderately deficient in protein were reported to increase food consumption in some (6–8), but not all (9, 10) studies. Notably, protein intake across 13 countries was found to be remarkably stable at ~16% of total calories (11), and even partial protein leverage caused by a reduction in protein intake was predicted to contribute to at least one-third of weight gain and the obesity epidemic (12). In contrast to other species, previous studies were unable to detect a hyperphagic response to mild [<25% lower crude protein (CP) than requirements] and moderate (25–50% lower CP than requirement) protein restriction in pigs. We and others showed that moderate protein restriction (12–14% CP) reduced feed intake in pigs (13–15), but slightly low protein diets (25% lower CP than requirement) did not change their energy intake (14, 16, 17). Reduced food intake in response to low protein diets (22% metabolizable energy) has been also reported in cats (18). However, severely low protein diets (50% lower CP than requirements) have been shown to increase the energy intake in pigs (19–21). A caveat is that most swine studies have primarily focused on improving production efficiency by supplementing essential amino acids to low protein diets, which adds complexity in interpreting the energy intake data. The resistance of species such as pigs to mount a hyperphagic response to mild to moderate dietary protein restriction, but showing an increased energy intake in response to severe protein restriction, is suggestive of differences in protein dilution threshold sensing by different species, which warrants further studies. Thus, gaining insights into the mechanisms of food intake regulation by low protein diets is important for developing effective prevention strategies for weight gain and improving feed efficiency.

### Mechanisms of Sensing Protein Insufficiency

The hepatic amino acid sensing and signaling mechanisms play an important role in detecting amino acid insufficiency to coordinate a systemic response to restore protein balance. A relative deficiency of dietary amino acids leads to accumulation of uncharged cytoplasmic tRNA that bind to general control non-derepressible (GCN2) which in turn phosphorylates eukaryotic translation initiation factorα (eIF2α) leading to activation of activating transcription factor 4 (ATF4) and CCAAT/Enhancer-binding protein homologous protein (CHOP) to inhibit protein synthesis and increase fibroblast growth factor-21 (FGF21) expression and secretion (22–25). Further, GCN2 through other intermediaries inactivates mTORC1 leading to dephosphorylation of 4E-binding protein (4EBP1) to inhibit protein synthesis (22, 26). Consistent with these studies that were mostly conducted *in vitro* and with amino acids, we (27) and others (28–30) showed that a similar pathway for sensing dietary protein deficiency also operates in the liver to upregulate hepatic FGF21 expression and secretion. Interestingly, we found that similar amino acid sensing pathways were also upregulated in the duodenum (31) suggesting that the intestine may detect protein deficiency prior to the liver, and/or that the intestinal sensing may serve to amplify the hepatic response to protein restriction. Independent of sensing by gut-associated tissues, amino acid deprivation causes a rapid anorexic response that is triggered by GCN2 signaling in the piriform cortex (32, 33).

### Mechanisms of Regulation of Food Intake by Low Protein Diets

**Figure 1:**
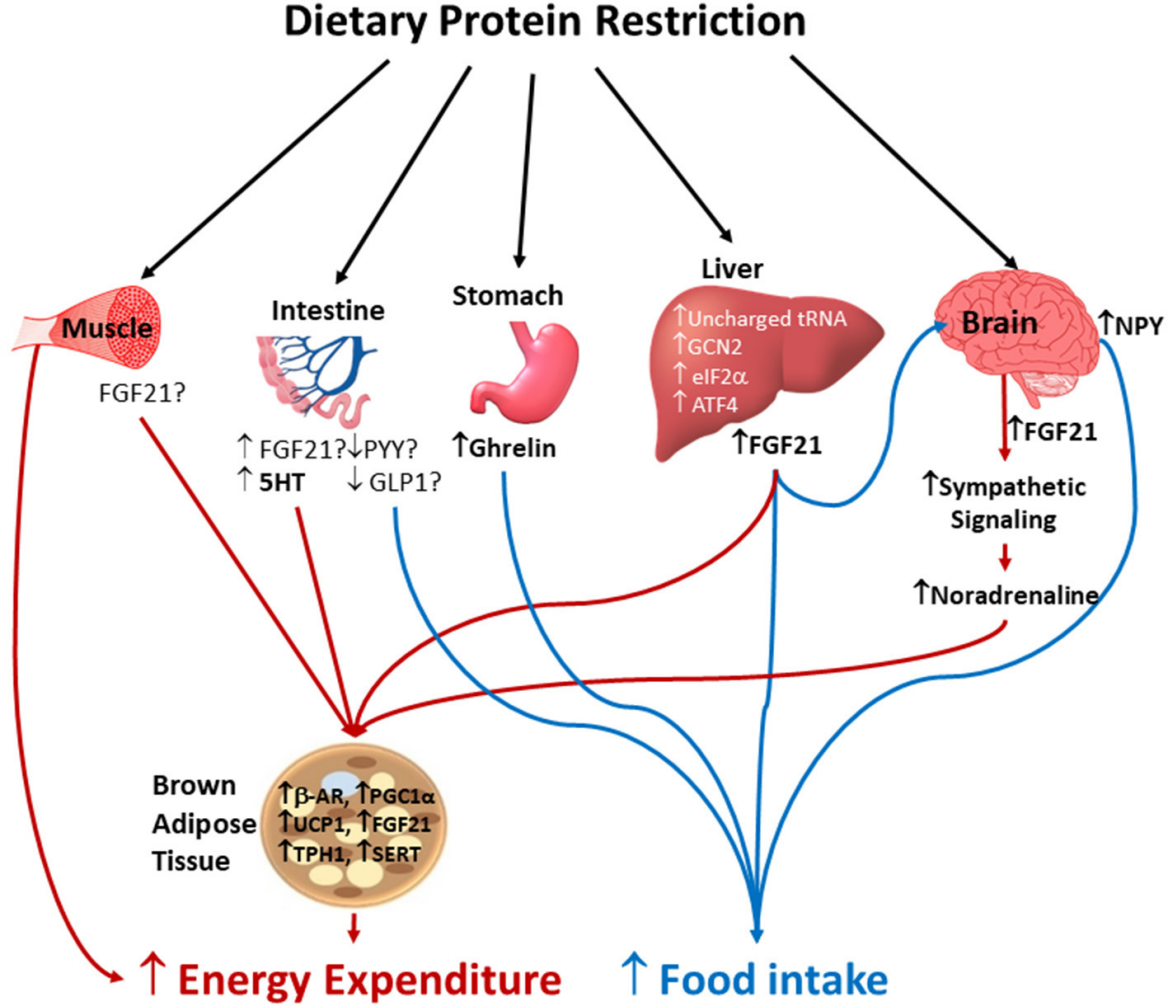
Potential mechanisms by which protein dilution alters energy intake and energy expenditure. Protein restriction concurrently increases energy intake and energy expenditure. Amino acids insufficiency is sensed by liver, small intestine, and brain by the involvement of unchanged tRNA, non-derepressible (GCN2), eukaryotic translation initiation factorα (eIF2α) and activating transcription factor 4 (ATF4) which results in hyperphagia to restore protein balance. The low protein induced hyperphagia is possibly mediated by increased circulating concentrations of ghrelin and fibroblast growth factor-21 (FGF21), and neuropeptide Y (NPY) expression in the hypothalamus. Whether reduced secretion of anorexigenic gut peptides such as peptide YY (PYY) and glucagon-like peptide 1 (GLP-1) contribute to increased energy intake following protein dilution is not completely known. The increased energy expenditure in response to protein restriction is mediated by multiple mechanisms including increased sympathetic flux and upregulation of thermogenic markers in brown adipose tissue (BAT), and muscle thermogenesis. Whether local FGF21 secreted by skeletal muscle, small intestine and BAT, and 5-hydroxytryptamine (5HT) signaling in BAT play a role in low protein induced thermogenesis via autocrine, paracrine and endocrine pathways remains unclear. B-AR, beta adrenergic receptors; UCP1, uncoupling protein-1; PGC-1α, proliferator-activated receptor gamma coactivator 1-alpha; TPH1, tryptophan hydroxylase 1; SERT, serotonin transporter.

Accumulating evidence indicates that moderate protein restriction in rodents (5–8% protein kcal) stimulates FGF21 secretion from the liver which acts on β-klotho receptors in the brain to promote hyperphagia (34–36) (Figure 1). We have also shown that such hyperphagic responses to protein dilution are a consequence of increased meal size in rodents (31). The hyperphagic responses to moderate protein dilution are also associated with reduction in circulating concentrations of leptin and IGF-1, increased plasma ghrelin, and upregulation of orexigenic neuropeptide Y transcripts in the rodent hypothalamus (27, 31, 37, 38). Although the role of anorexigenic lower gut peptides such as peptide YY and glucagon-like peptide-1 (GLP-1) in high-protein induced hypophagia is well-documented in rodents (39–41) and humans (42), we were unable to detect a graded insufficiency in circulating concentrations of these and other anorectic gut hormones (e.g., gastric inhibitory polypeptide and amylin) in rats (27, 31) that might explain the hyperphagia with protein dilution. It is unknown whether sensitivity to hypophagic effects of these satiety hormones is impaired with dietary protein restriction. However, birds fed with low protein diets were found to have a higher occurrence of GLP-1-immunoreactive cells in the ileum (43). Though the hyperphagic responses to protein dilution are consistently observed in a no-choice condition (27–31, 36), when protein restricted rodents are given a choice of macronutrients, they tend to prefer protein (8, 34, 44) with reduced preference for carbohydrates (45, 46). Similarly, in humans, the fMRI BOLD responses to food cues were greater in reward areas such as orbitofrontal cortex and striatum under low protein conditions (7). In contrast to mild protein restriction, severe protein restriction or depletion (<5% protein kcal) leads to profound hypophagia with a rapid reduction in both meal size and number in rats (31). Such aversive responses are due to rapid sensing of the amino acid imbalance by the anterior piriform cortex where the GCN2–eIF2α pathway is activated to repress protein synthesis, together with activation of glutamatergic outputs to the hypothalamus to cause anorexia (47). The relative importance of peripheral and central sensing and signaling mechanisms that regulate food intake in response to varying degrees of dietary protein restriction across species remains to be resolved.

## Regulation Of Energy Expenditure By Low Protein Diets

### Effects of Low Protein Diets on Energy Expenditure

Protein or total amino acid restriction increases thermogenesis in rodents (27, 29, 31, 35, 36, 48–61). An increased EE was also reported in pigs fed with severely low protein diets (19–21). In one study, pigs maintained equal body weight when fed protein deficient diets with high or low energy content suggestive of enhanced EE in these groups (62). We also showed that moderate reduction of dietary protein results in an increased EE in early weeks of study in young pigs (13, 14). Similarly, protein restricted diets have also been shown to acutely increase EE in humans (63–65). Overall, increased EE and subsequently reduced food efficiency may contribute to reduced weight gain and lean mass during protein restriction (27, 36, 56, 58, 66).

Given the concurrent increase in EE and energy intake in response to protein restriction (27, 51, 56, 60), and as increased EE is generally considered a compensatory response to hyperphagia (67–69), one may question whether the enhanced EE with protein restriction is a consequence of increased energy intake. Leveraging effect of dietary protein content on energy intake has been reported previously (49) suggesting that low protein induced EE could be considered as a primary response to dietary protein content. Recently, we showed that protein restricted rats sustain an enhanced EE in the absence of hyperphagia, which is suggestive of an energy intake independent pathway for low protein induced thermogenesis (31). Likewise, others have shown that low protein induced EE occurs independent of hyperphagia (35) and that energy intake changes as a secondary response to compensate for the enhanced EE (70). Therefore, it appears that increased EE in response to protein restriction is partly related to enhanced basal metabolic rate component of total EE (53), although the contribution of basal metabolic rate and diet-induced thermogenesis was reported to be negligible (71). An increased spontaneous motor activity was reported in mice fed with low protein diets (71), which does not seem to be related with the overall activity level (69, 71). Further research is required to better understand the effect of low protein diets with variable dietary carbohydrate and fat contents on different components of total EE.

Due to high carbohydrate content of low protein diets (13, 14, 27, 48, 49, 51, 53–56, 60, 61) an increased EE in response to such diets could be the result of either high carbohydrate or low protein content. In an independent study, using obesity-prone rats we showed that low protein diets with fixed carbohydrate, but variable fat contents increased EE (31), similar to low protein-high carbohydrate diets (27). A greater EE response to protein dilution was also observed in humans regardless of dietary carbohydrate and fat content (65). Altogether, these studies suggest that enhanced EE is likely a primary response to protein restriction rather than carbohydrate or lipid content, or energy intake; however, further studies are required to assess the contribution of dietary carbohydrate and fat content to enhanced EE under protein restriction.

### Mechanisms of Regulation of Energy Expenditure by Low Protein Diets

Despite intense efforts to gain insights into the hyperphagic responses driven by protein dilution, the underlying mechanisms of changes in EE received less scrutiny. The thermogenic effects of low protein diets have been associated with (i) increased sympathetic flux to brown adipose tissue (BAT) via β-adrenergic receptor (β-AR) signaling (50, 51, 55–57, 72, 73), as well as stimulation of thermogenesis in white adipose tissue (74) and muscle (27), (ii) FGF21 and mitochondrial uncoupling protein-1 (UCP1) mediated mechanisms (27, 29, 31, 36, 70, 75) and (iii) serotonergic signaling (27, 76) (Figure 1).

The BAT plays an important role in diet-induced thermogenesis (77–79). An increased sympathetic influx to BAT appears to be essential for the thermogenic effects of low protein diets (50–52, 55, 56). This involves release of noradrenaline from postganglionic sympathetic nerve terminals and subsequent interaction of noradrenaline with β-AR, in particular β3-AR in BAT (80–83). We and others showed that the transcripts of adrenergic signaling and thermogenic markers including β2 and β3-AR, peroxisome proliferator-activated receptor gamma coactivator 1-alpha and UCP1 were increased in BAT of rats fed with protein deficient diets (27, 57). We also showed that low protein induced EE is attenuated following administration of propranolol, a β1 and β2-AR antagonist (27, 31). This suggests that low protein induced EE is mediated by sympathetic signaling.

Fibroblast growth factor-21 is released in response to nutrient deficiency and stimulates browning of white adipose tissue to regulate adaptive thermogenesis (53, 74). Infusion of FGF21 increases EE and core body temperature in rodent models (84–86). Using FGF21 deficient rodent models, the effect of protein restricted diets on basal and cold-induced EE have been shown to be FGF21 dependent (35, 61, 87, 88). Although hepatic FGF21 seems to stimulate BAT thermogenesis via an endocrine pathway (29, 30, 36, 74, 89), the FGF21 expression and secretion in BAT is also increased by sympathetic stimulation (72, 90). We and others showed that plasma FGF21 concentrations and transcripts in liver, small intestine, skeletal muscle and BAT were increased when rodents are fed with low protein diets (27, 31, 36, 53, 60, 75, 91). Further, methionine and leucine restriction increases FGF21 expression particularly in liver and circulation (92–94). Dietary protein restriction also increases circulating FGF21 concentrations in humans (36, 61, 63–65). FGF21 appears to be produced in variety of organs including skeletal muscle in response to cellular stress triggered by various stimuli (95–99). Whether FGF21 derived from BAT, muscle and other organs reaches the circulation and contributes to low protein induced thermogenesis via endocrine pathway remains unclear, but it appears that FGF21 most likely mediates the low protein induced EE through endocrine, paracrine and autocrine signaling. Further, FGF21 signaling in glutamatergic neurons of the ventromedial hypothalamus appears to be essential for the increase in EE with dietary protein dilution (45). The roles of peripheral and central FGF21 sensing and signaling mechanisms in the thermogenic effects of dietary protein restriction remains to be further delineated.

The role of serotonergic neurons in regulation of thermogenesis and BAT activity has been previously documented (100, 101). In particular, central serotonin signaling is crucial for the activity of BAT and thermoregulation (102–104). Serotonergic signaling also appears to be associated with low protein induced EE. We showed that the mRNA abundance of tryptophan hydroxylase 1, an enzyme involved in biosynthesis of serotonin, and serotonin transporter is increased in the BAT of rats fed with low protein diets which might suggest a paracrine or autocrine control of low protein enhanced EE by local 5-hydroxytryptamine (5-HT or serotonin). The VO_2_ and EE were shown to be decreased by administration of a non-selective 5-HT receptor antagonist, metergoline (76) and 5-HT3 receptor antagonist, ondansetron (27) in rats fed low protein diets. This is suggestive of higher serotonergic tone in rats fed with protein restricted diets. Whether both central and peripheral serotonergic signaling are equally essential for the thermogenic effects of low protein diets remains to be studied.

Dietary protein restriction results in reduced concentration of most essential amino acids in the circulation, which play a role in metabolic adaptations to protein deficient diets. Among the amino acids studied, methionine (60, 105–108), tryptophan (41), and leucine (109) restriction have been shown to enhance EE. This is suggestive of the importance of amino acid profile and protein quality in regulation of thermogenesis. We showed that methionine restriction can partly recapitulate the total amino acid restriction-induced EE (48). This increase in EE in response to methionine restriction has been linked with greater secretion of hepatic FGF21 (92, 94, 110), upregulation of UCP1 in BAT (105, 107) and increased sympathetic signaling (48, 108). We and others using pharmacological (i.e., chemical sympathectomy and propranolol) and genetic (i.e., β3 receptor knockout mice) tools, showed that sympathetically driven enhanced EE in response to methionine restriction is mediated by β-AR (48, 108). Similarly, increased EE following tryptophan and leucine restrictions is mediated through sympathetic system and upregulation of UCP1 in BAT as well as FGF21 (41, 93, 111, 112). Whether deficiency of other essential amino acids play a role in the higher thermogenic effects of low protein diets, and the underlying pathways and organs involved, warrants further investigation.

## Conclusions And Future Implications

Dietary protein restriction orchestrates a whole organismal physiological response to stimulate energy intake and EE. The low protein induced thermogenesis is largely driven by dietary protein content and less likely by the concurrent hyperphagia. Though the liver and brain appear to be the primary sites for sensing protein deficiency, the coordination of these tissues with intestinal sensing mechanisms to promote hyperphagia and thermogenesis remains poorly understood. Although liver driven FGF21 seems essential for stimulating EE in response to protein or amino acid restricted diets, the role of local FGF21 synthesized and released from BAT, skeletal muscle and gut in the thermogenic responses to low protein diets remains to be determined. Further, the roles of systemic and local amino acid concentrations, as well as sympathetic and serotonergic signaling in brain, adipose and muscle tissues to coordinate intake and expenditure responses to dietary protein restriction remains largely unexplored. A deeper understanding of the neuroendocrine mechanisms by which dietary protein dilution modulates energy balance may lead to the development of novel strategies for preventing and treating obesity and associated comorbidities in humans, as well as for improving production efficiency in domestic animals.

## Author Contributions

AP and PC wrote the article and agree to be accountable for the content of the work. All authors contributed to the article and approved the submitted version.

## Conflict of Interest

The authors declare that the research was conducted in the absence of any commercial or financial relationships that could be construed as a potential conflict of interest.
